# cGMP interacts with tropomyosin and downregulates actin-tropomyosin-myosin complex interaction

**DOI:** 10.1186/s12931-018-0903-z

**Published:** 2018-10-12

**Authors:** Lihui Zou, Junhua Zhang, Jingli Han, Wenqing Li, Fei Su, Xiaomao Xu, Zhenguo Zhai, Fei Xiao

**Affiliations:** 1 The MOH Key Laboratory of Geriatrics, Beijing Hospital, National Center of Gerontology, Beijing, 100730 People’s Republic of China; 2 Department of Pathology, Beijing Hospital, National Center of Gerontology, Beijing, 100730 People’s Republic of China; 3 Department of Respiratory and Critical Care Medicine, Beijing Hospital, National Center of Gerontology, Beijing, 100730 People’s Republic of China; 4 Department of Respiratory and Critical Care Medicine, China-Japan Friendship Hospital, Beijing, 100029 People’s Republic of China

**Keywords:** Cyclic guanosine monophosphate, Tropomyosin, Actin, Myosin, Interaction

## Abstract

**Background:**

The nitric oxide-soluble guanylate cyclase-cyclic guanosine monophosphate (NO-sGC-cGMP) signaling pathway, plays a critical role in the pathogenesis of pulmonary arterial hypertension (PAH); however, its exact molecular mechanism remains undefined.

**Methods:**

Biotin-cGMP pull-down assay was performed to search for proteins regulated by cGMP. The interaction between cGMP and tropomyosin was analyzed with antibody dependent pull-down in vivo. Tropomyosin fragments were constructed to explore the tropomyosin-cGMP binding sites. The expression level and subcellular localization of tropomyosin were detected with Real-time PCR, Western blot and immunofluorescence assay after the 8-Br-cGMP treatment. Finally, isothermal titration calorimetry (ITC) was utilized to detect the binding affinity of actin-tropomyosin-myosin in the existence of cGMP-tropomyosin interaction.

**Results:**

cGMP interacted with tropomyosin. Isoform 4 of *TPM1* gene was identified as the only isoform expressed in the human pulmonary artery smooth muscle cells (HPASMCs). The region of 68-208aa of tropomyosin was necessary for the interaction between tropomyosin and cGMP. The expression level and subcellular localization of tropomyosin showed no change after the stimulation of NO-sGC-cGMP pathway. However, cGMP-tropomyosin interaction decreased the affinity of tropomyosin to actin.

**Conclusions:**

We elucidate the downstream signal pathway of NO-sGC-cGMP. This work will contribute to the detection of innovative targeted agents and provide novel insights into the development of new therapies for PAH.

## Background

PAH is a serious yet poorly understood disease characterized by elevated pulmonary artery pressure and vascular resistance and may ultimately result in right heart failure and death. The burden of small pulmonary arteries increases due to the contraction of vascular smooth muscle cells, which plays a crucial role in regulating of pulmonary vascular resistance and in the development of PAH [1]. However, the regulatory mechanism of the contraction of intrapulmonary arteries is poorly understood, and further investigations, especially those on molecular biomarkers, are essential for decoding the underlying pathogenic mechanism of PAH.

The NO-sGC-cGMP pathway is closely associated with PAH. cGMP exerts its biological activity mainly via three main groups of down-stream targets: cGMP-dependent protein kinases, cGMP-gated cation channels, and phosphodiesterases. Malfunctions in NO production, sGC activity, and cGMP degradation cause pulmonary arterial vasodilatation through abnormal vascular smooth muscle cell proliferation and platelet aggregation [2].

Our previous study found that the activation of NO-sGC-cGMP pathway led to abnormal expression of multiple genes including SERPINB2, MMP1, GREM1, and IL8 [3]. We have demonstrated that SERPINB2, also known as PAI-2, suppresses the proliferation and migration of cultured HPASMCs and speeds their apoptosis [4]. The mRNA level of PAl-2 in peripheral blood cells of PAH patients was significantly decreased and the expression of PAl-2 was up-regulated by sGC activator. PAI-2 can be used as a potential biomarker for vascular remodeling of PAH [5]. MMP1 encodes matrix metalloprotease-1, a member of the matrix metalloprotease (MMP) family, which are the major proteases involved in tissue remodelling of the extracellular matrix by degrading all types of matrix components. Therefore, NO-sGC-cGMP pathway may assuage or reverse the vascular remodeling in PAH by regulating genes including PAl-2 and MMP1.

However, besides these downstream-regulated genes, little is known about the interaction proteins of cGMP. As a second messenger in cells, cGMP hardly acts as isolated species while performing its functions in vivo. In our current study, we performed the Biotin-cGMP pull-down assay to search for the proteins interacted and regulated by cGMP with a human pulmonary artery smooth muscle cell line. Fortunately, we found that cGMP interacted with tropomyosin, a key factor that regulates smooth muscle contraction. Tropomyosin regulated the productive interaction between myosins and actin by shifting its position on the actin filament. The isothermal titration calorimetry (ITC) assay was then utilized to explore the fast actin-tropomyosin-myosin complex alteration after the activation of NO-sGC-cGMP pathway. We demonstrated that actin-tropomyosin-myosin interaction was downregulated in the existence of cGMP-tropomyosin interaction competition and caused the down-regulation of muscle contraction. All these findings demonstrate the functional interaction between cGMP and tropomyosin, which is involved in the regulation of muscle contraction.

## Methods

### Cell culture and treatment

HPASMCs (ScienCell Research Laboratories, Carlsbad, USA), identified by immuefluorescent antibodies to α-smooth muscle actin and desmin, were isolated from human pulmonary arteries [6]. Smooth muscle cell medium (ScienCell Research Laboratories, Carlsbad, USA) supplemented with 2% fetal bovine serum and 1% smooth muscle cell growth supplement was utilized. HPASMCs were then maintained at 37 °C humidified atmosphere of 5% carbondioxide. P3 ~ P5 cells were used for experiments. The exponentially growing HPASMCs were treated with 100 μM of 8-Br-cGMP (Sigma–Aldrich Corp., St. Louis, USA) for 24 h.

### Biotin-cGMP pull-down assay

10 μM of Biotin-cGMP and the negative control Biotin (Biolog life science institute, Bremen, Germany) were nurtured in a mix of whole HPASMC cell lysates at 4 °C for 6 h with gentle rotation. Biotin-cGMP fusion protein and Biotin protein were then attached to 100 μl of streptavidin magnetic beads at 4 °C for 6 h. After being gently washed by lysis buffer for 3–5 times gently to reduce nonspecific binding, beads were incubated with 50 μl of glycine elution buffer (100 mM, PH 2.5) for 10 min. Then, 10 μl of NH_4_HCO_3_ neutralization buffer was added to each elution collection tube to neutralize the pH of the contents upon elution. The elution fractions were boiled in sodium dodecyl sulphate-polyacrylamide gel electrophoresis (SDS–PAGE) loading buffer and analyzed by silver staining method or immune blotting analysis. After having been excised, protein bands were subjected to in-gel trypsin digestion and analysed by mass spectrometry.

### RNA extraction, reverse transcription, and real-time PCR

Total RNAs were extracted from HPASMCs with Trizol reagent (Invitrogen Life Technologies, Carlsberg, USA), following the manufacturer’s protocol (Invitrogen). The reverse transcription was performed with the total RNAs (2 μg) and the Moloney Murine Leukemia Virus reverse transcription kit (Invitrogen Life Technologies, Carlsberg, USA).

Real-time PCR was performed to quantify *TPM1* gene, and expression of the internal control gene *ACTIN* was determined by iQ5 Multicolor Real-time PCR Detection System (Bio-Rad Laboratories, Hercules, CA, USA). The PCR reaction used 9.9 μl of distilled water, 12.5 μl of 2X SYBR® Premix Ex TaqTMII, 0.8 μl each for primers, and 1 μl of DNA template, constructing a total volume of 25 μl. The two-step PCR protocol was performed using SYBR® Premix Ex TaqTM II (Takara Biotech Co., Dalian, China) following the manufacturer’s instruction. It included one cycle of 95 °C for 30 s, 40 cycles of 95 °C for 5 s, and 60 °C for 30 s. Each reaction was run in triplicate. The Ct values for relative quantification of gene expression were used to determine the *TPM1* expression levels. The PCR primers for *TPM1* were as follows: 5’-GCTGCAGAGGATAAGTACTC-3′ (forward), 5’-CATGTTGTTTAACTCCAGT-3′ (reverse); *β*-*ACTIN* 5’-GGCGGCACCACCATGTACCCT-3′ (forward), 5’-AGGGGCCGGACTCGTCATACT -3′ (reverse). The PCR primers for *TPM1* gene isoforms were as follows: Primer 1: 5′- CGGTCGCCCCCTTGGGAAAG -3′ (forward), 5′- GCTTGTCGGAAAGGACCTTGATCTC -3′ (reverse); Primer 2: 5′- AGTGAGAGAGGCATGAAAGTC -3′ (forward), 5′- AATTTAGTTACTGACCTCTCCGCA -3′ (reverse); Primer 3: 5′- CGGGCTGAGTTTGCGGAGAGG -3′ (forward), 5′- AAGGAATGGAAGTCTCGGAAGA -3′ (reverse).

### Protein isolation and Western blot

Cells were collected and rinsed with PBS solution, and the protein was extracted by using the protein lysis buffer (including 1% NP-40, 1 μg/ml aprotinin, 1 μg/ml leupeptin, and 100 μg/ml PMSF). The protein concentration was assessed with a Bio-Rad DC Protein Assay (Bio-Rad, Hercules, USA). Then, 50 μg of proteins were used for sodium dodecyl sulphate-polyacrylamide gel electrophoresis. Subsequently, the proteins were transferred to nitrocellulose membranes by electro blotting (Millipore Corp., Boston, USA). Membranes were blocked in 5% milk, washed with PBST (PBS with 0.1% Tween), and incubated with 1:1000 anti-tropomyosin antibody (cell signaling technology, Danvers, USA) at 4 °C for 12 h and 0.4 μg/ml anti-actin antibody (ZSBIO, Beijing, China) at 4 °C for 1 h. After the washing and incubation with 0.08 μg/ml horseradish peroxidase-conjugated anti-IgG antibody (ZSBIO, Beijing, China) at 4 °C for 1 h in 5% milk, the membranes were washed for three times and subjected to the enhanced chemiluminescent reagents (Millipore Corp., Boston, USA) for the distinction of protein bands.

### Immunofluorescence assay

Cells were grown and treated with 8-Br-cGMP on cover slips. Cultured cells were affixed with methanol at room temperature for 20 min. After being washed with PBS for three times, cover slips were blocked for 60 min in BSA blocking buffer, then incubated with diluted primary antibody at room temperature for 2 h. Cells were rinsed in PBS three times (for 5 min each), then incubated with diluted fluorochrome-conjugated secondary antibody (ZSBIO, Beijing, China) at room temperature for 1 h in dark. After stained with DAPI and stored with mounting medium, the samples were examined under confocal microscope.

### Isothermal titration calorimetry assay

Interaction analysis among actin, myosin, and tropomyosin were performed at 298 K (degree kelvin) with a MicroCal Isothermal Titration Calorimeter ITC200 instrument (Malvern, England). The investigations were performed according to a strictly standardized protocol [7–9]. The highly purified proteins were prepared by Sephadex G-50 column with automated protein separation chromatography system (Jinhua Inc., Shanghai, China). Typically, the syringe was filled with 60 μL of protein solution at a concentration of 0.05 μM that subsequently titrated into a 300 μL protein with the concentration of 0.005 μM in the cell. There were twenty injections of 2 μL each with a spacing of 120 s between injections employed to allow the system to reach the equilibrium. Heat generated by titrant dilution was tested by a control experiment, titrating into buffer alone, in uniform arragements. On the basis of a self-sufficient binding sites model, the MicroCal-Origin 7.0 software was applied to fit the integrated heat data of the titrations by a non-linear least-squares minimization algorithm.

The fitting parameters were ΔH (reaction enthalpy change in cal mol^− 1^), ΔS (the changes in entropyin cal mol^− 1^), K (the inverse of equilibrium binding constant in M^− 1^), and N (molar ratio of the proteins in the complex). Δ H value is equal to the constant pressure heat of reaction. The positive value of Δ H means heat absorption and the negative value represents heat release. Entropy is a measure of system disorder. ΔS associates with the change in the amount of substance before and after reaction in the system.Gibbs free energy (ΔG) had a relationship with ΔH that was ΔG = ΔH-TΔS = -RTlnKa (*R* = 8.314 J mol^− 1^ K^− 1^, *T* = 298.15 K), which was applied to calculate the reaction energy eventually. All the ΔG obtained by calculation testified the spontaneous reactions in our experiments.

### Statistical analysis

All data attained in triplicate independent experiments were assessed via SPSS statistics 20.0 and GraphPad prism 5 (GraphPad software, Inc., La Jolla, USA). Data are showed as mean ± SD. Statistical comparisons were carried out with the student *t* test for investigating two-group data. A *P* value of < 0.05 was considered statistically significant

## Results

### cGMP directly interacted with tropomyosin

**Fig. 1:**
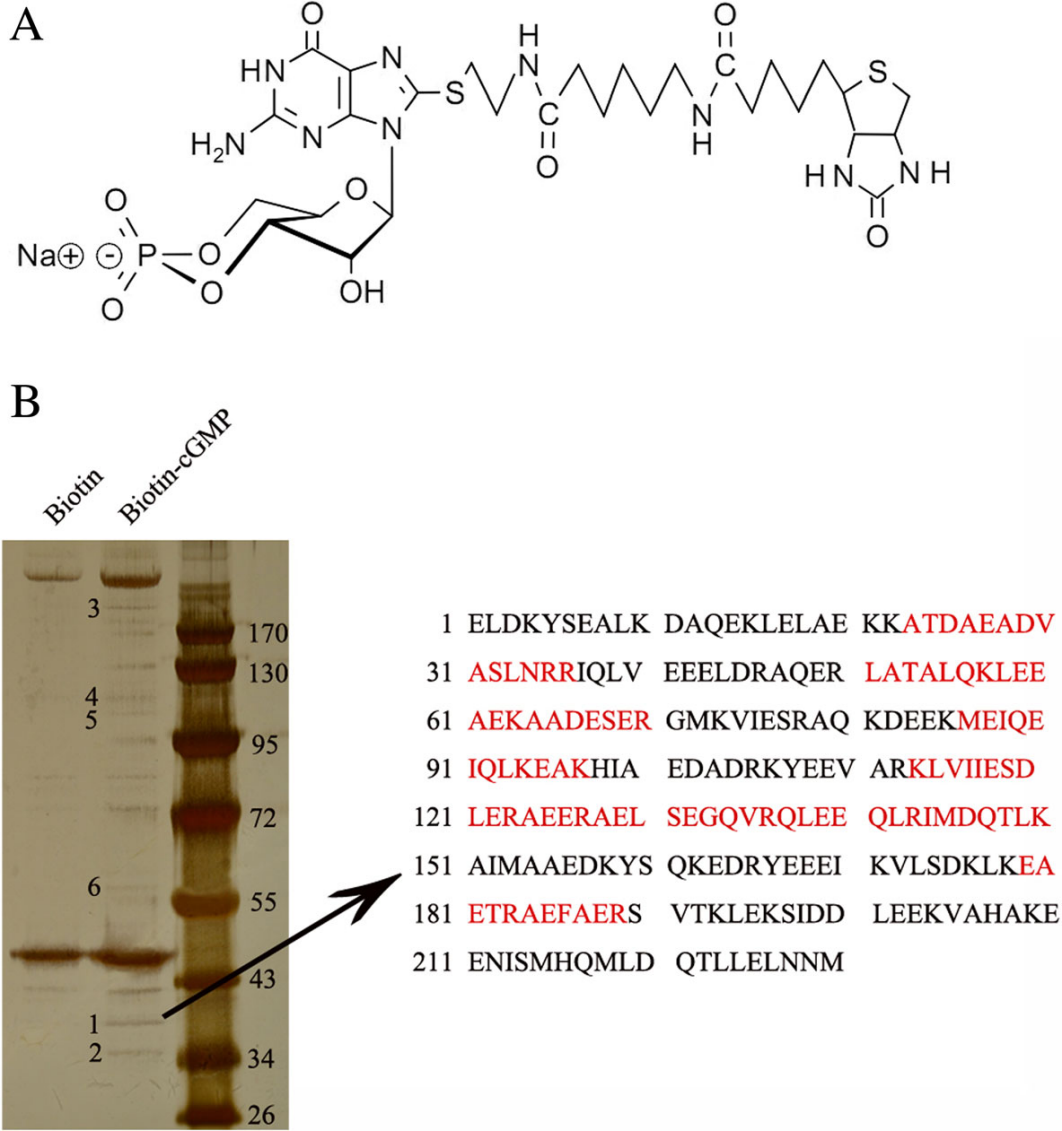
cGMP directly interacts with tropomyosin. (**a**) The chemical structural formula of 8-[Biotin]-AET-cGMP. Biotin is connected to the 8-position of cyclic GMP via a 11 atom spacer. (**b**) Characterization of interaction proteins with cGMP in vitro. Cell extractions from HPASMCs were pulled down with the biotin-cGMP, subjected to SDS–PAGE and visualized by silver staining. The bands associated with cGMP were pointed out by the numbers (bands 1–6) and identified by mass spectrometry. The peptides of the band 1 belong to tropomyosin are indicated by red letters

Biotin-cGMP is a cGMP analog that has similar properties with cGMP (Fig. 1a). The bands characteristic to cGMP are presented in Fig. 1b. Compared with the negative control biotin, six bands were uniquely associated with cGMP (line 2). The protein bands associated with cGMP were then extracted, digested with trypsin and analysed by mass spectrometry. The result showed that 5 peptides of the band 1 belonged to the protein tropomyosin, which was involved in the regulatory system of actin-myosin interaction. The other bands corresponded to the protein keratin and some uncharacterized proteins with protein scores less than 66, suggesting the observed match was a random event.

### **Isoform 4 was expressed in the HPASMCs**

**Fig. 2:**
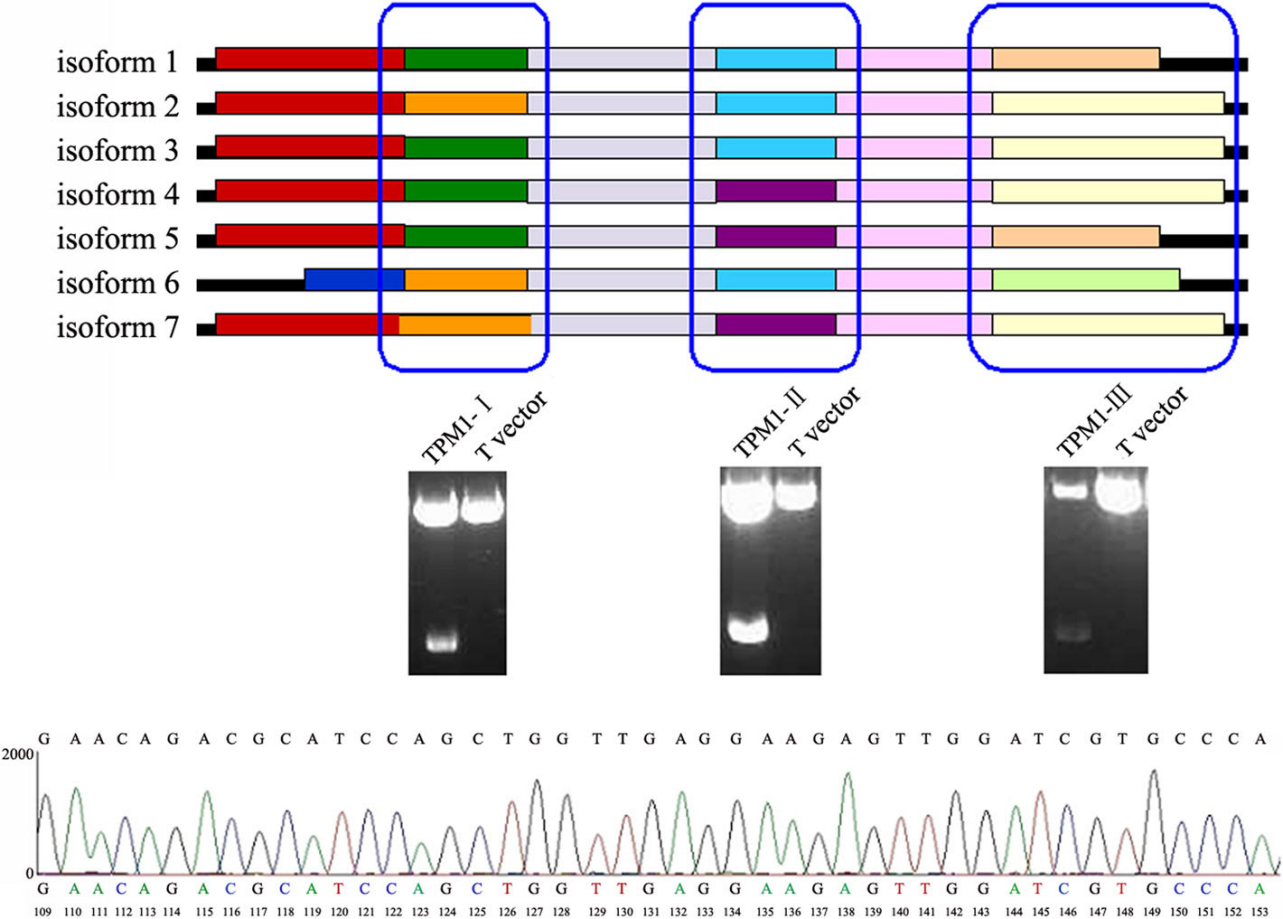
Analysis of the isoforms of *TPM1* gene in the HPASMCs. The homologous analysis of the seven transcripts of *TPM1* gene in the HPASMCs. The blue boxes indicate the non-conserved sequ’ences

In order to determine which isoform was expressed in the HPASMCs, we performed three PCR using primers designed on the basis of the three difference sequences (Fig. 2). The products of PCR were cloned into pGEM®-T expression vector and sequenced, which was found to be in full compliance with isoform 4. The partial base sequences were shown in Fig. 2. The sequencing results also indicated that isoform 4 was the only isoform expressed in the HPASMCs.

### cGMP specifically bound to tropomyosin

**Fig. 3:**
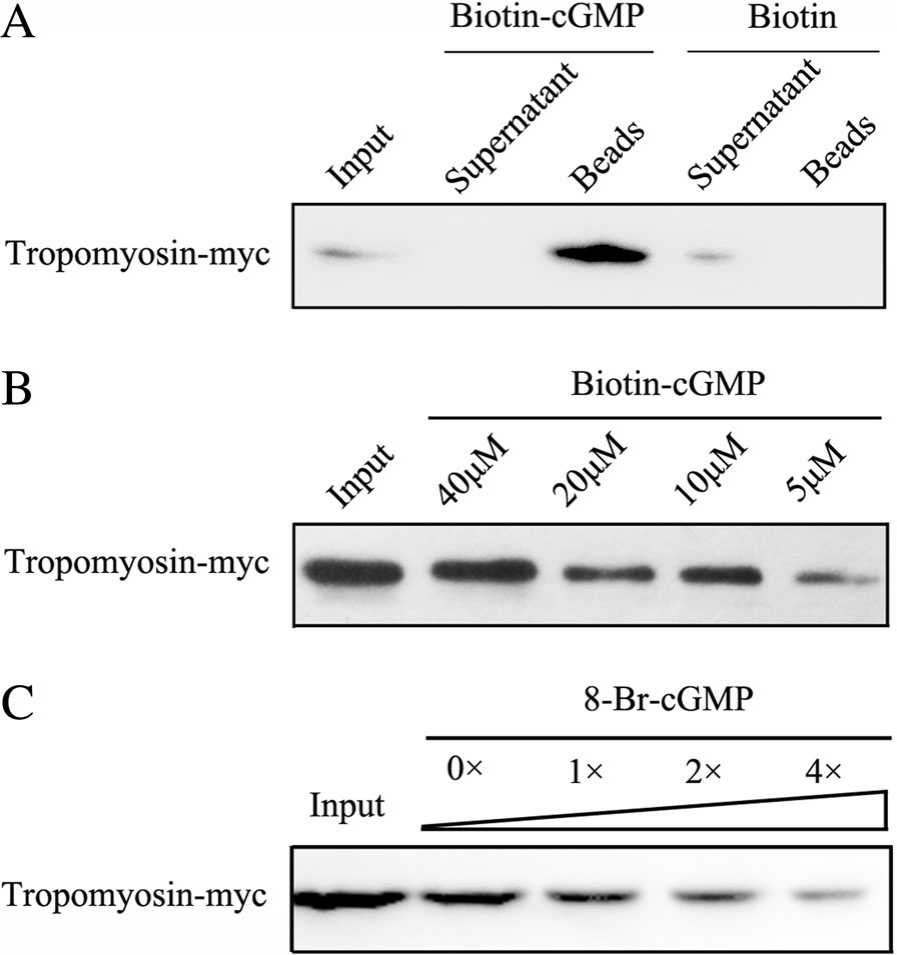
Identification of the association of cGMP and tropomyosin. (**a**) Proteins from Tropomyosin-Myc transfected HPASMC extracts were pulled down with the biotin-cGMP, separated in SDS-PAGE gels, and analyzed by immunoblotting with specific antibody to Myc. (**b**) Different amounts of biotin-cGMP were incubated with a mixture of protein extraction, and the eluted proteins were also subjected to SDS–PAGE. Antibodies against Myc were utilized in the Western blot analysis. (**c**) Various amounts of 8-Br-cGMP were added to compete with biotin-labeled cGMP in interacting with Tropomyosin-Myc

To further confirm the interaction between cGMP and tropomyosin in vivo, antibody dependent pull-down was performed. Isoform 4 of *TPM1* gene was amplified and cloned into pcDNA3.1(−)-cMyc vector, then transfected into cultured HPASMCs. The biotinylated cGMP and the biotin alone were both incubated with HPASMC cell lysates. Tropomyosin-Myc was validated with antibody against Myc in the biotin-cGMP pull-down protein complexes, but not in the biotin pull-down protein complexes (Fig. 3a). Meanwhile, the interaction between cGMP and tropomyosin was alterable in the existence of different amounts of biotin-cGMP (Fig. 3b). Furthermore, competition assay was performed to detect the specificity of cGMP and tropomyosin interaction. The interaction between cGMP and tropomyosin was competed by 8-Br-cGMP (non-biotinylated cGMP) in a dose-related manner (Fig. 3c).

### Region of 68-208aa was necessary for the interaction between tropomyosin and cGMP

**Fig. 4:**
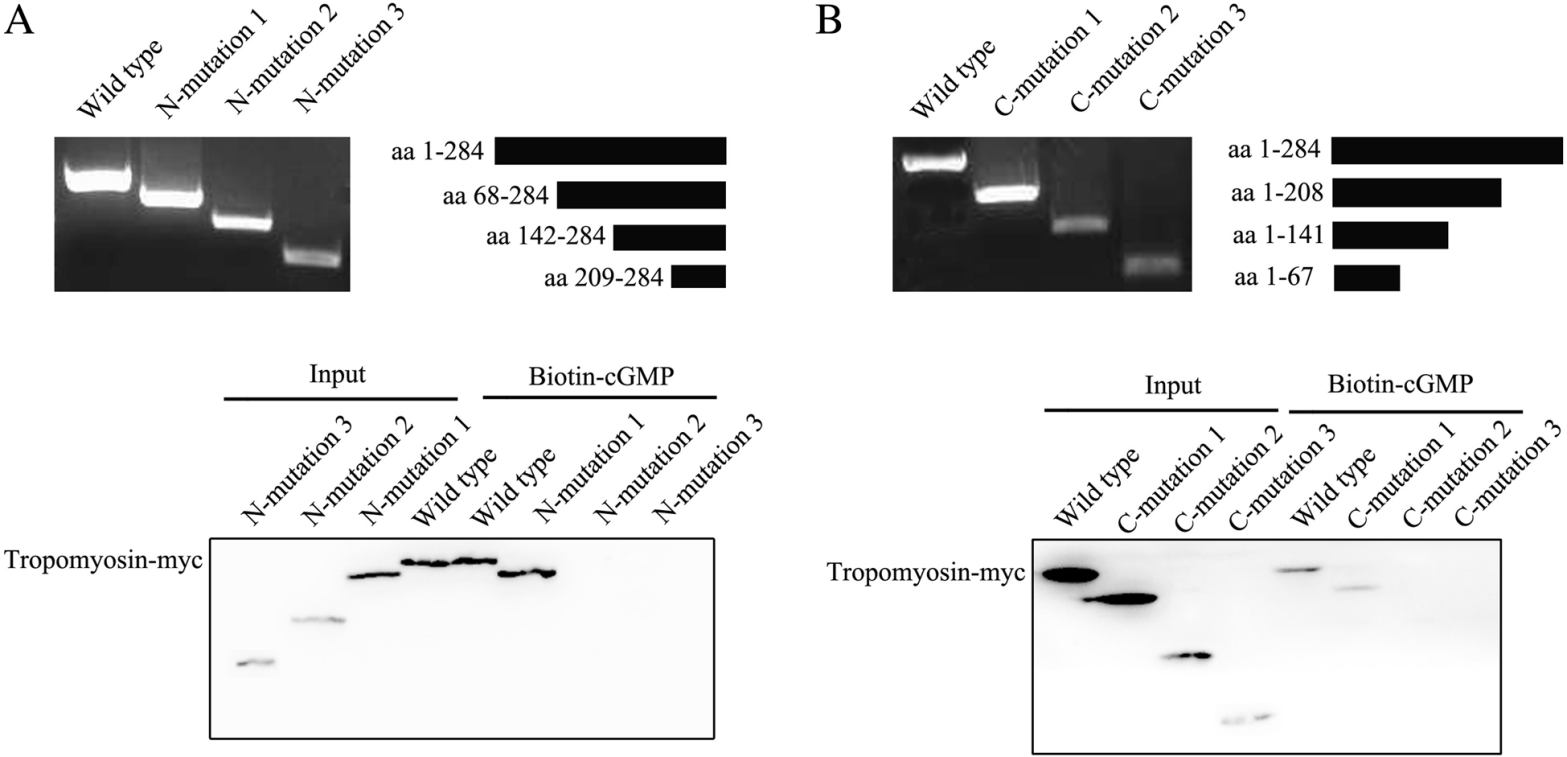
The schematic representations of tropomyosin fragments and their interaction with cGMP. (**a**) Tropomyosin fragments aa 1–284, aa 68–284, aa 142–284, and aa 209–284 cloned into pcDNA3.1(−)-cMyc expression vector. Proteins from tropomyosin fragments transfected HPASMC extracts were pulled down with the biotin-cGMP, separated in SDS-PAGE gels and analyzed by immunoblotting with specific antibody to Myc. (**b**) As described in (**a**), aa 1–284, aa 1–208, aa 1–141, and aa 1–67 fragments of tropomyosin were transfected into HPASMCs for 24 h respectively and the cellular lysates were immunoprecipitated with anti-Myc antibody

To explore the binding sites of tropomyosin interacted with cGMP, tropomyosin fragments (aa 1–284, aa 68–284, aa 142–284, aa 209–284, aa 1–208, aa 1–141, and aa 1–67, respectively named as wild type, N-mutation 1, N-mutation 2, N-mutation 3, C-mutation 1, C-mutation 2, and C-mutation 3) were inserted into pcDNA3.1(−)-cMyc vector and then transfected into cultured HPASMCs. These vectors could express Myc-fused protein in cells. Antibody-dependent pull-down was repeated. As shown in Fig. 4a and b, wild type, N-mutation 1, and C-mutation 1 were detected in the immunocomplexes. In contrast, N-mutation 2, N-mutation 3, C-mutation 2, and C-mutation 3 were absent from the immunocomplexes with antibodies against Myc. Collectively, these results indicated that there was a cGMP binding site at aa 68–208 of the tropomyosin protein.

### cGMP had no effect on TPM1 expression and subcellular localization

**Fig. 5:**
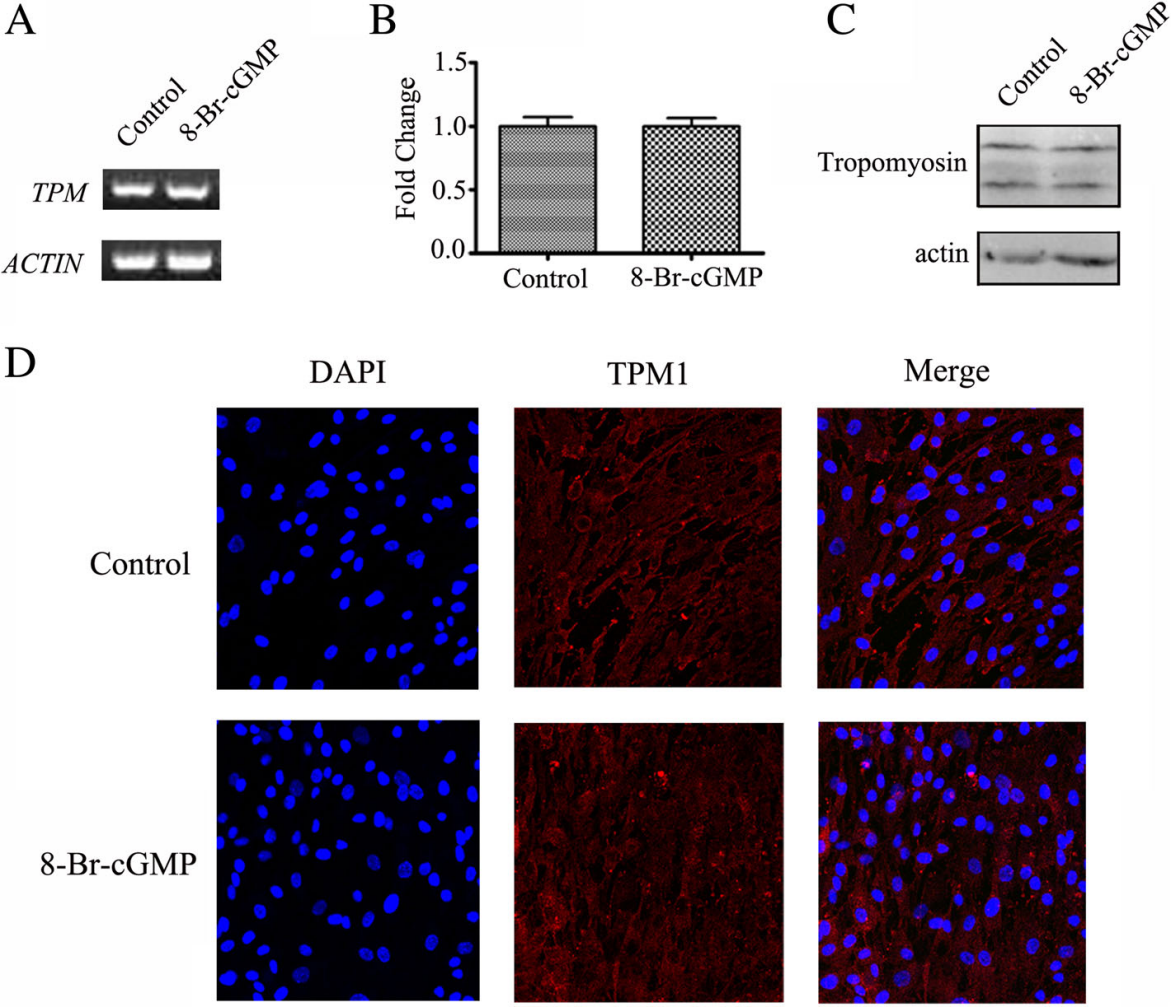
cGMP has no effect on *TPM1* expression and subcellular localization. (**a**) HPASMCs were treated with 100 μM of 8-Br-cGMP. After 8 h, electrophoretic analysis of the expression of *TPM1* was performed by reverse transcription PCR. (**b**) HPASMCs were treated with 100 μM of 8-Br-cGMP. The expression levels of *TPM1* were normalized to *ACTIN* levels after Real-time PCR. The data were obtained from three individual experiments and are expressed as mean ± SD. (**c**) HPASMCs were treated with 100 μM of 8-Br-cGMP for 24 h. The protein expression levels of tropomyosin were analyzed by Western blotting with specific antibodies. (**d**) HPASMCs were treated with 100 μM of 8-Br-cGMP, fixed and stained with antibodies to tropomyosin, and then incubated with FITC-conjugated IgG. Cellular nuclei were stained with DAPI. All the experiments were performed in triplicate and were repeated at least three times

HPASMCs were induced by 8-Br-cGMP (100 μM), *TPM1* expression was not significantly dysregulated after 8-Br-cGMP induction compared to the control cells (Fig. 5a, b, and c). cGMP has no effect on tropomyosin subcellular localization after 8-Br-cGMP induction compared to the control cells (Fig. 5d).

### Analysis of actin-tropomyosin-myosin interactions by ITC

ITC is mainly applied to explore molecular binding reactions and provides the binding constant, the molar ratio of the proteins complex, and the enthalpy change (ΔH) of the interaction [10–12]. We utilized ITC to detect the binding affinity of actin-tropomyosin-myosin. However, there were numerous non-specific binding activities caused by electrostatic interactions in each assay. To minimize this, the sodium chloride concentration of the buffer in the ITC assays was decreased from 50 mM to 5 mM.

**Fig. 6:**
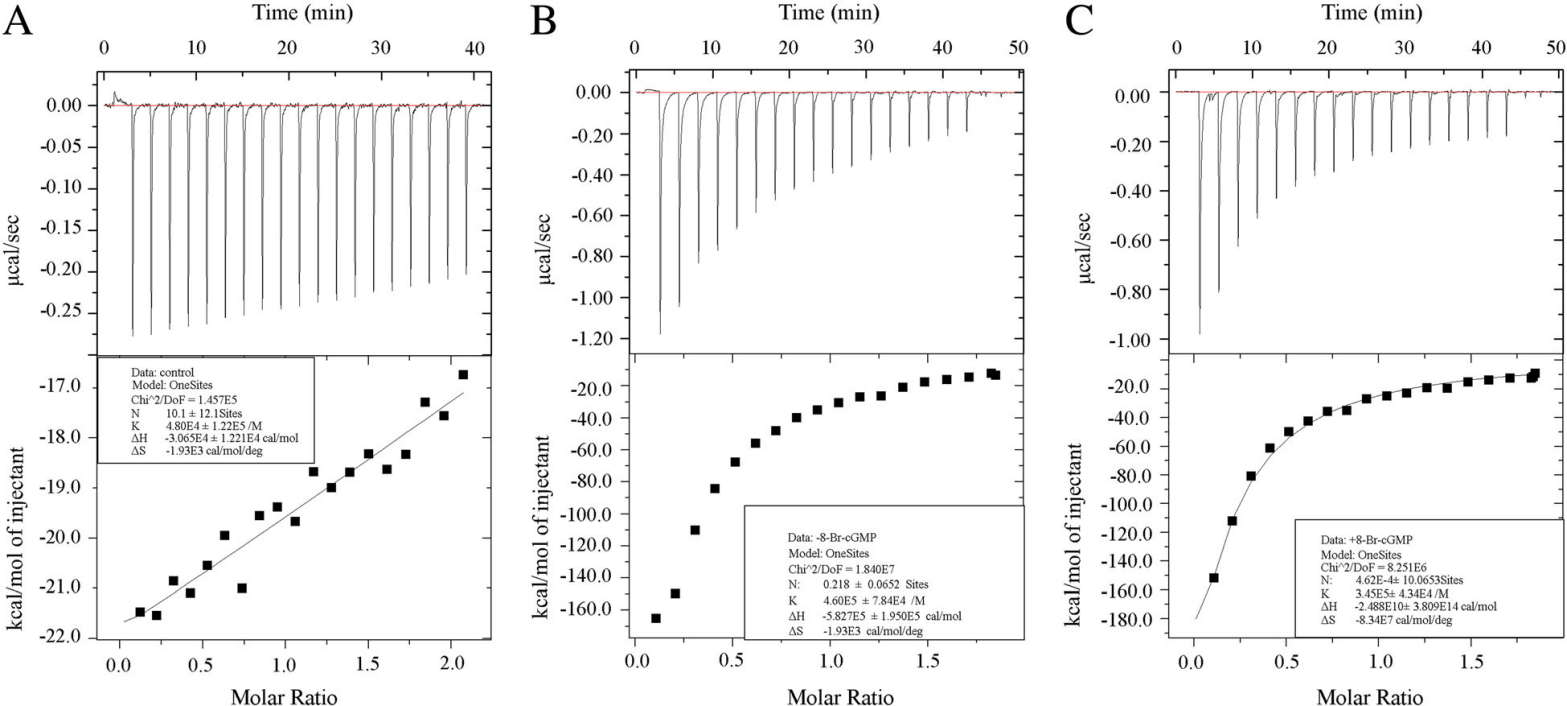
Analysis of actin-tropomyosin-myosin interactions by ITC. Raw titration data showed the thermal effect of protein interaction. (**a**) Diluted buffer was titrated into a calorimetric cell containing actin and myosin mixture. (**b**) Tropomyosin was titrated into a calorimetric cell containing actin and myosin mixture. (**c**) Tropomyosin mixtured with 8-Br-cGMP (100 μM) were titrated into a calorimetric cell containing actin and myosin mixture. The normalized heat of interaction was gained in all cases by integrating the raw data and subtracting the heat of ligand dilution into the buffer alone. The grey line represents the best fit obtained by a non-linear least-squares procedure based on an independent binding sites model. Experiments were repeated at least three times

Under the experimental status, the binding affinity of tropomyosin to actin-myosin was significantly up-regulated compared to the control group (Fig. 6a and b). However, the binding affinity was significantly down-regulated after adding 8-Br-cGMP, with K value changed from (4.60 × 10^5^ ± 7.84 × 10^4^) to (3.45 × 10^5^ ± 4.34 × 10^4^) (Fig. 6b and c).

Table 1 shows the binding constants and thermodynamic parameters. There was a stronger affinity of actin, tropomyosin and myosin without treatment of 8-Br-cGMP proved byΔG compared to the experimental group with 8-Br-cGMP, which is consistent with the meaning of another parameter K value. Significant difference was found between the experimental group without 8-Br-cGMP and the group with 8-Br-cGMP (*P* < 0.05), suggesting a distinct obstruction of the formation of actin-tropomyosin-myosin complex.

**Table 1:** Binding constants and thermodynamic parameters of actin-tropomyosin-myosin systems detected with ITC

Interaction	T	K(/mol)	ΔH (kcal/mol)	ΔS(kcal/mol)	ΔG(kcal/mol)
Control/Actin+Myosin	298.15	4.80 × 10^4^ ± 1.22 × 10^5^	−30.70 ± 12.20	−0.08	− 30.70 ± 12.20
Tropomyosin/Actin+Myosin	298.15	4.60 × 10^5^ ± 7.84 × 10^4^	−5.83 × 10^5^ ± 1.95 × 10^5^	−1.93	− 5.82 × 10^5^ ± 1.95 × 10^5^
Tropomyosin/Actin+Myosin+ 8-Br-cGMP	298.15	3.45 × 10^5^ ± 4.34 × 10^4^	−2.49 × 10^7^ ± 3.81 × 10^11^	−8.34 × 10^4^	−3.43 × 10^4^ ± 3.81 × 10^11^

## Discussion

Smooth muscle cell is the most important regulator of the blood vessel contraction in the body. Smooth muscle contraction can be caused by several physiochemical agents. The ectopic regulation of NO production, sGC activity and cGMP degradation contributes to the development of pulmonary vasorelaxation, which is closely related to the effect of smooth muscle cell. Riociguat (BAY 63–2521), the sGC stimulator, has a dual effect on PAH by promoting pulmonary vasorelaxation and reducing fibrosis [13]. However, the molecular mechanism by which NO-sGC-cGMP pathway causes pulmonary vasorelaxation remains undefined.

In this study, by performing Biotin-cGMP pull-down assay and ITC analysis, we found that cGMP interacted with tropomyosin, which lead to the structural and functional alterations in the actin-tropomyosin-myosin complex. Tropomyosin, as a coiled-coil molecule which associates head-to-tail to form super-helical polymers, has 284 aa in length with a molecular mass of about 33 kDa [14]. Mammals utilize *TPM1*, *TPM2*, *TPM3*, and *TPM4* to express more than 40 tropomyosin isoforms by alternative splicing [15]. The functions of isoforms are variables determined by the timing and tissue specificity of gene expression, mRNA, and protein localization. For example, tropomyosin 4 [16] expression is enhanced and tropomyosin 1 [17] expression is altered in smooth muscle cells shifting from a contractile to a synthetic phenotype. Tropomyosin 6 is a relatively late marker for smooth muscle maturation in both mice and human beings [18]. Thus, the diverse functions of tropomyosin isoforms have offered an impressive mechanism to illustrate the variation of actin-myosin interaction in different intracellular compartments [19]. We performed PCR using primers designed based on the non-conserved sequences and found that isoform 4 was the only isoform expressed in our HPASMCs.

It is becoming increasingly clear that muscle contraction is controlled by the sliding filament mechanism, whose central feature is the interplay of myosin with actin filaments [20]. The heads of myosin in the thick filament bind to actin in the thin filament (made of actin and tropomyosin) as induced by some physiochemical signals. Tropomyosin plays a crucial role in the regulating the interaction between actin and myosin and altering the sliding of myosin filaments, thus serving as a regulator of muscle contraction [21].

In our current study, we demonstrated that there was an interaction between cGMP and tropomyosin, and the cGMP binding site was aa 68–208 of the tropomyosin protein. According to the 3-D protein structure from the Protein Data Bank (PDB, https://www.rcsb.org/), aa 98–233 of tropomyosin is the key fragment that regulates the interaction between myosin and actin. Thus, cGMP and tropomyosin interaction may compete to the binding site of tropomyosin and actin, finally leading to the decrease of the interaction between myosin and actin. This was in accordance with the “steric blocking mechanism” that the migration of tropomyosin into the center of the filament groove increases the interaction between the myosin head and actin [20].

Furthermore, smooth muscle contraction is set up in motion by the discharge of Ca^2+^ [22–24]. ATPase is also needed during the contraction [25]. Therefore, Ca^2+^ and ATPase can be added to the mixure for ITC assay, permitting the myosin head to interact with the actin filament and cause contraction.

We also found in our study that, although cGMP interacted with tropomyosin, it had no effect on tropomyosin expression and subcellular localization. The expression of tropomyosin can be regulated by other factors; for example, miR-21 can down-regulate *TPM1* to inhibit tumor growth, and thus miR-21 functions as an oncogene [26]. Another second messenger cAMP also negatively regulates the mRNA level of tropomyosin in cultured rat vascular smooth muscle cells [27]. We assume that there are two main ways of tropomyosin to exert its functions: one way is the activated pathway, in which expression and localization are adjusted by unique signals or modifications; and the other way is the physical interaction, i.e. tropomyosin exerts crucial functions via its interactions with other critical cellular proteins.

This preliminary study clearly demonstrated an interaction of cGMP with tropomyosin, although we still have no data on cGMP-tropomyosin interaction in vivo. In addition to investigations on the pulmonary artery pressure after the increase of cGMP level in tropomyosin 1 knock-out mice, and hPASMC contraction after cGMP increase and TPM1 knockdown, we also will detect the cGMP-tropomyosin interaction in PAH mice. Also, the structure of cGMP and actin-tropomyosin-myosin complex need be further defined with methods such as x-ray diffraction and electron microscopy.

## Conclusions

In summary, our study delineated a physical interaction between cGMP and tropomyosin, that might contribute to the alterations of the structure and function of the actin-tropomyosin-myosin complex and finally bridge the suppressive effect of pulmonary vasorelaxation. These findings shed new light on molecular mechanism by which NO-sGC-cGMP pathway acts as a down-regulation factor of muscle contraction in the pathogenesis of PAH.

## Abbreviations

HPASMC: Human pulmonary artery smooth muscle cell;
ITC: Isothermal titration calorimetry
NO-sGC-cGMP: Nitric oxide-soluble guanylate cyclase-cyclic guanosine monophosphate
PAH: Pulmonary arterial hypertension

## References

[1] Schermuly RT, Ghofrani HA, Wilkins MR, Grimminger F (2011). Mechanisms of disease: pulmonary arterial hypertension. Nat Rev Cardiol.

[2] Baliga RS, Macallister RJ, Hobbs AJ (2013). Vasoactive peptides and the pathogenesis of pulmonary hypertension: role and potential therapeutic application. Handb Exp Pharmacol.

[3] Zou L, Xu X, Zhai Z, Yang T, Jin J, Xiao F (2016). Identification of downstream target genes regulated by the nitric oxide-soluble guanylate cyclase-cyclic guanosine monophosphate signal pathway in pulmonary hypertension. J Int Med Res.

[4] Zhang S, Zou L, Yang T, Yang Y, Zhai Z, Xiao F (2015). The sGC activator inhibits the proliferation and migration, promotes the apoptosis of human pulmonary arterial smooth muscle cells via the up regulation of plasminogen activator inhibitor-2. Exp Cell Res.

[5] Zou L, Zhang S, Xu X, Xiao F, Zhai Z (2016). Expression of PAI-2 mRNA in peripheral blood leucocytes and regulation by sGC activator in pulmonary hypertension. Zhonghua Yi Xue Za Zhi.

[6] Wang G, Liu X, Meng L, Liu S, Wang L, Li J (2014). Up-regulated lipocalin-2 in pulmonary hypertension involving in pulmonary artery SMC resistance to apoptosis. Int J Biol Sci.

[7] Wylie T, Garg R, Ridley AJ, Conte MR (2017). Analysis of the interaction of Plexin-B1 and Plexin-B2 with Rnd family proteins. PLoS One.

[8] Sun Q, He J, Yang H, Li S, Zhao L, Li H (2017). Analysis of binding properties and interaction of thiabendazole and its metabolite with human serum albumin via multiple spectroscopic methods. Food Chem.

[9] Hands-Taylor KL, Martino L, Tata R, Babon JJ, Bui TT, Drake AF (2010). Heterodimerization of the human RNase P/MRP subunits Rpp20 and Rpp25 is a prerequisite for interaction with the P3 arm of RNase MRP RNA. Nucleic Acids Res.

[10] Rajarathnam K, Rosgen J (2014). Isothermal titration calorimetry of membrane proteins - progress and challenges. Biochim Biophys Acta.

[11] Pierce MM, Raman CS, Nall BT (1999). Isothermal titration calorimetry of protein-protein interactions. Methods.

[12] Loh W, Brinatti C, Tam KC (2016). Use of isothermal titration calorimetry to study surfactant aggregation in colloidal systems. Biochim Biophys Acta.

[13] Stasch JP, Pacher P, Evgenov OV (2011). Soluble guanylate cyclase as an emerging therapeutic target in cardiopulmonary disease. Circulation.

[14] Ruiz-Opazo N, Nadal-Ginard B (1987). Alpha-tropomyosin gene organization. Alternative splicing of duplicated isotype-specific exons accounts for the production of smooth and striated muscle isoforms. J Biol Chem.

[15] Gunning P, O'Neill G, Hardeman E (2008). Tropomyosin-based regulation of the actin cytoskeleton in time and space. Physiol Rev.

[16] Abouhamed M, Reichenberg S, Robenek H, Plenz G (2003). Tropomyosin 4 expression is enhanced in dedifferentiating smooth muscle cells in vitro and during atherogenesis. Eur J Cell Biol.

[17] Girjes AA, Keriakous D, Cockerill GW, Hayward IP, Campbell GR, Campbell JH (2002). Cloning of a differentially expressed tropomyosin isoform from cultured rabbit aortic smooth muscle cells. Int J Biochem Cell Biol.

[18] Vrhovski B, McKay K, Schevzov G, Gunning PW, Weinberger RP (2005). Smooth muscle-specific alpha tropomyosin is a marker of fully differentiated smooth muscle in lung. J Histochem Cytochem.

[19] Gunning PW, Schevzov G, Kee AJ, Hardeman EC (2005). Tropomyosin isoforms: divining rods for actin cytoskeleton function. Trends Cell Biol.

[20] Behrmann E, Muller M, Penczek PA, Mannherz HG, Manstein DJ, Raunser S (2012). Structure of the rigor actin-tropomyosin-myosin complex. Cell.

[21] Parry DA, Squire JM (1973). Structural role of tropomyosin in muscle regulation: analysis of the x-ray diffraction patterns from relaxed and contracting muscles. J Mol Biol.

[22] Ueda K, Kimura-Sakiyama C, Aihara T, Miki M, Arata T (2013). Calcium-dependent interaction sites of tropomyosin on reconstituted muscle thin filaments with bound myosin heads as studied by site-directed spin-labeling. Biophys J.

[23] Gergely J (1998). Molecular switches in troponin. Adv Exp Med Biol.

[24] Li MX, Wang X, Sykes BD (2004). Structural based insights into the role of troponin in cardiac muscle pathophysiology. J Muscle Res Cell Motil.

[25] Sweeney HL, Houdusse A (2010). Structural and functional insights into the myosin motor mechanism. Annu Rev Biophys.

[26] Zhu S, Si ML, Wu H, Mo YY (2007). MicroRNA-21 targets the tumor suppressor gene tropomyosin 1 (TPM1). J Biol Chem.

[27] Ohara O, Nakano T, Teraoka H, Arita H (1991). cAMP negatively regulates mRNA levels of actin and tropomyosin in rat cultured vascular smooth muscle cells. J Biochem.

